# Communication issues between caregivers and patients with concealment of advanced-stage cancer: A qualitative study

**DOI:** 10.51866/oa.574

**Published:** 2024-09-10

**Authors:** Sanhapan Wattanapisit, Apichai Wattanapisit, Pornnapat Laksanapiya, Arunee Tipwong

**Affiliations:** 1 MD, MSc, MRCFPT, Palliative Care Unit, Thasala Hospital, Nakhon Si Thammarat, Thailand. Email: th.sanhapan@gmail.com; 2 MD, FRCFPT, Academic Fellowship, (Family Medicine), Department of Clinical Medicine, School of Medicine, Walailak University, Nakhon Si Thammarat, Thailand.; 3 Family Medicine Clinic, Walailak, University Hospital, Nakhon Si Thammarat, Thailand.; 4 MD, MRCFPT, Palliative Care Unit, Thasala Hospital, Nakhon Si Thammarat, Thailand.; 5 MD, MA, FRCFPT, Academic, Fellowship (Family Medicine), Department of Social Medicine, Surat Thani Hospital, Surat Thani, Thailand.

**Keywords:** Cancer, Communication, Palliative care

## Abstract

**Introduction:**

Communication is a key element of palliative care. The concealment of advanced-stage diseases is a communication challenge. This study aimed to explore the patterns and difficulties in communication regarding the concealment of advanced-stage cancer between caregivers and patients. Methods: This qualitative study employed an interpretive phenomenological approach and was conducted at a district hospital in Thailand. Semi-structured in-depth interviews were performed to collect data from caregivers (i.e. family members) of patients regarding the concealment of advanced-stage cancer. The data analysis followed an inductive thematic approach.

**Results:**

Ten in-depth interviews were conducted among the caregivers of patients aged 57-97 years. Four themes emerged: (i) reasons for concealing the diagnosis and prognosis (personality of patients and concerns about negative effects), (ii) communication patterns between caregivers and patients (communicating symptoms/signs instead of the diagnosis/prognosis and distorting information), (iii) difficulties and challenges in maintaining concealment (feelings of guilt, hesitation in sharing the information and suspicion of patients’ awareness of their diagnosis/prognosis) and (iv) communication support from healthcare professionals (avoiding informing patients about their diagnosis/prognosis, supporting decision-making and disclosing the information).

**Conclusion:**

The concealment of advanced-stage cancer is perceived as an appropriate communication approach among some caregivers. Communicating information about advanced-stage cancer is dynamic. Some caregivers and families consider disclosing the information in the future. Healthcare professionals can support communication throughout care. Future studies should focus on decision-making and communication processes for better handling of information concealment or the conspiracy of silence in palliative care.

## Introduction

Palliative care is an approach that aims to improve the quality of life and relieve suffering among patients with incurable and life-threatening conditions.^1^ It comprises three major components: (i) treatments of physical and psychological symptoms, (ii) spiritual care and (iii) communication.^2^ Communication in palliative care requires specific skills. One of the important issues is diagnostic and prognostic disclosure to patients.

In some cultures and beliefs, families and caregivers wish to conceal information about incurable diagnoses and poor prognoses because the truth may cause negative impacts on patients, such as discouragement, emotional stress and premature deaths.^3-6^ Previous studies have revealed that only 30%-78% of patients perceive real information about their diagnoses and prognoses.^7-10^ However, a systematic review on prognostic disclosure indicated both negative and positive impacts on patients’ quality of life.^11^

Patients with terminal illnesses have their own right and dignity to desire treatment options.^12^ However, a lack of truth-telling or information concealment leads to difficulties in further communication and care among healthcare providers.^13^ In addition, the conspiracy of silence wherein family members and healthcare providers avoid providing real information to patients can cause negative consequences (e.g. insufficient treatment for abnormal symptoms or neglect of spiritual care).^14^

To the best of the authors’ knowledge, the concealment of diagnosis and prognosis among patients with advanced-stage cancer based on their families’ wishes is common in Thailand. Cancer is taboo in the Thai context and several Asian cultures.^15-18^ Palliative care service in the authors’ setting has dealt with this communication challenge, hindering the improvement of the quality of care provided. However, some families and caregivers wish to conceal information about the diagnosis and prognosis from patients. This raises an issue regarding communication between caregivers and patients.

This study aimed to explore the patterns and difficulties in communication regarding the concealment of advanced-stage cancer between caregivers and patients. Exploring caregivers’ viewpoints would help in gaining a better understanding and fostering effective communication among healthcare providers, caregivers, families and patients in palliative care.

## Methods

### Design

This qualitative study employed an interpretive phenomenological approach, aiming to understand the experiential phenomenon.^19^ The ontological assumption was relativism (reality is different for each person and subjective), and the epistemological assumption was subjectivism (knowledge depends on real-world experiences).^20^ The Standards for Reporting Qualitative Research were adopted to ensure transparency of the methodologies and manuscript writing.^21^

### Context and participants

This study was conducted at a 300-bed district hospital in southern Thailand, where palliative care services cover outpatient, inpatient and home care. In the hospital, the palliative care team consists of trained family physicians (physicians who have completed a short-course palliative training or a master’s degree in the field of palliative care), nurses and a pharmacist.

Participants were caregivers concealing advanced-stage cancer from patients. Patients were not included as participants in this study to avoid disclosing the information without family approval. Caregivers were invited to participate in the study when they were 18 years old or above and when their patients had received at least one consultation with the palliative care team. Caregivers who were non-family members and/or had communication barriers (e.g. cognitive impairment or dementia) were excluded.

Eligible caregivers were contacted by one of the authors. The author verbally and briefly explained the study details. When caregivers agreed to participate in the study, the author asked them to suggest a convenient time for an interview. Participation in the study was voluntary. Refusal to participate did not affect patient care. The author provided a participant information sheet and allowed caregivers to enquire about unclear information before providing written informed consent.

### Data collection

Data about caregivers (i.e. age, sex, relationship to the patient and duration of caregiving) and patients (i.e. age, sex, religion, type of cancer, duration of advanced-stage cancer, duration of palliative care and Palliative Performance Scale (PPS) score) were collected and recorded in a hardcopy form. Generally, the PPS score ranges from 0% (death) to 100% (full ambulation and healthy).^22^ The first author (SW: a family physician with a master’s degree related to palliative care), who was experienced in qualitative research, conducted all in-depth interviews in the Thai language following the interview guide. The interview questions were developed based on the study objectives of exploring patterns and difficulties in communication (Table 1). Probing questions were asked to clarify and expand participants’ answers. During each interview, a digital audio recorder was used to record the interview when participants permitted. The number of interviews was based on data saturation (i.e. when no new data emerged).

**Table 1:** Semi-structured interview guide (translated version).

No.	Question
1	What is your role in taking care of the patient?
2	Why is the patient not informed about the advanced or incurable stage of cancer?
3	How do you communicate with the patient about supportive care, such as treatment goals, medication usage and their symptoms?
4	How do you communicate with the patient when (s)he asks about the diagnosis, disease stage or test results?
5	Which communication issues with the patient do you find difficult to address or emotionally challenging?
6	What is your opinion if the patient is aware of the advanced stage of cancer or the stage that cannot be cured?
7	What role do you think the medical team/hospital should play in supporting communication between caregivers and patients?

### Data analysis

All audio recordings were transcribed verbatim and typed in Microsoft Word (Microsoft, Redmond, WA, USA). The first author (SW) checked the transcripts for accuracy. The transcripts were imported to a qualitative analysis software (NVivo, release 1.7.1, QSR International, Victoria, Australia). The age of each caregiver and patient was presented in ranges, and the identifiers of caregivers were coded to maintain anonymity and confidentiality.

The data were analysed using an inductive thematic approach.^23,24^ Two authors (SW and AW: an academic family physician with experience in qualitative research) read the transcripts to familiarise themselves with the data. The first author (SW) generated initial codes and thematic maps. Another author (AW) cross-checked the thematic maps. Subsequently, the two authors defined the final themes and sub-themes. Any discrepancy in the data analysis was resolved via discussion and consensus among the research team. Themes and selected quotations were translated from Thai to English by the first author (SW) at the time of manuscript writing. The translation was verified and approved by all authors.

## Results

Ten in-depth interviews were conducted. Each interview took 20-34 minutes to complete. Most caregivers were women (n=9; 90%) and were caregiving from a few days to about 2 years. The majority were daughters or wives (n=8; 80%) of the patients with advanced-stage cancer. Among the 10 patients, who were aged 57-97 years, six were men, and four were women. Most patients were diagnosed with advanced-stage lung and colorectal cancers (n=6; 60%), and the PPS score was 20%-60% (Table 2).

**Table 2:** Caregiver and patient characteristics and codes.

Code	Caregiver information				Patient information						
	Age	Sex	Relationship	Duration of caregiving	Age range	Sex	Religion	Type of cancer	Duration of advanced-stage cancer	Duration of palliative care	PPS score
PI	<60 years	Female	Daughter	2 years 2 months	81-90 years	Male	Buddhism	Lung cancer	2 years 2 months	2 days	50%
P2	≥60 years	Female	Wife	2 years 2 months	71-80 years	Male	Buddhism	Oesophageal cancer	4 days	4 days	30%
P3	≥60 years	Female	Daughter	5 years	91-100 years	Female	Islam	Kidney cancer	5 days	4 days	40%
P4	<60 years	Female	Wife	20 days	51-60 years	Male	Buddhism	Colon cancer	20 days	7 days	20%
P5	<60 years	Female	Daughter	12 years	81-90 years	Male	Islam	Lung cancer	24 days	7 days	20%
P 6	<60 years	Female	Daughter	4 days	81-90 years	Female	Islam	Rectal cancer	4 days	3 days	30%
P7	<60 years	Female	Daughter	8 years	81-90 years	Male	Islam	Rectal cancer	11 months	11 months	60%
P8	<60 years	Female	Granddaughter-in-law	1 year	81-90 years	Female	Buddhism	Lung cancer	1 month	1 day	30%
P9	<60 years	Female	Daughter	10 days	81-90 years	Female	Buddhism	Liver cancer	10 days	3 days	20%
P10	<60 years	Male	Son	2 years	71-80 years	Male	Buddhism	Leukaemia	2 days	2 days	30%

P, participant; PPS, Palliative Performance Scale

**Figure 1:**
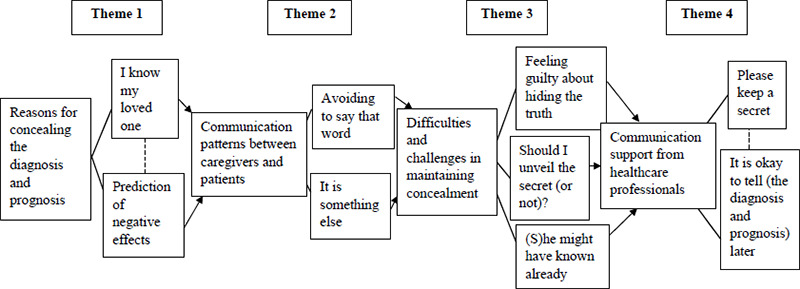
Conceptual summary of the themes and sub-themes.

The relevant codes, sub-themes and four major themes were identified: (i) reasons for concealing the diagnosis and prognosis, (ii) communication patterns between caregivers and patients, (iii) difficulties and challenges in maintaining concealment and (iv) communication support from healthcare professionals (Table 3). Figure 1 conceptualises the themes and sub-themes.

**Table 3:** Summary of the data codes and themes.

Code (n=time cited)	Sub-theme	Theme
Advanced care planning (n=5) Avoidance of truth-telling (n=4) Belief (n=3) Caregiver’s perception (n=27) Difficulty (n=6) Doubt about truth-telling (n=4) Family’s concern (n=6) Healthcare provider’s communication (n=8) Living will (n=8) Need from healthcare provider (n=7) Patient’s action (n=9) Patient’s experience (n=2) Patient’s perception (n=24) Pattern of communication (n=37) Personality (n=15) Reason to conceal (n=16)	I know my loved one Prediction of negative effects	Reasons for concealing the diagnosis and prognosis
	Avoiding to say that word It is something else	Communication patterns between caregivers and patients
	Feeling guilty about hiding the truth Should I unveil the secret (or not)? (S)he might have known already	Difficulties and challenges in maintaining concealment
	Please keep a secret It is okay to tell (the diagnosis and prognosis) later	Communication support from healthcare professionals

***Theme 1: Reasons for concealing the diagnosis and prognosis***

The caregivers perceived that concealing the diagnosis and prognosis from patients is appropriate. The main reasons for protecting patients from this information were based on the patients’ personality and concerns about negative effects.

*Sub-theme 1.1:1 know my loved one*

Some caregivers mentioned that they were familiar with their patients’ personality. Anxiety and overthinking were susceptible characteristics that influenced the caregivers’ and family’s decision to conceal information about the diagnosis and prognosis.

*‘He is unable to accept. I look after him, and I know he is. He looks strong but sensitive. When he is in pain, he cries. When I took care of my mom, she was patient and calm. He [my dad] cries loudly all the time’.*

(P5, daughter, caregiver of an 81-to-90-year-old man with lung cancer, Islam)

*It is... Her children do not want her to know because she has an anxious personality. If she did not have this disease, she would be anxious about her children. They all are adults. She thinks about other people, children’.*

(P6, daughter, caregiver of an 81-to-90-year-old woman with rectal cancer, Islam)

*Sub-theme 1.2: Prediction of negative effects*

The caregivers anticipated that revealing the diagnosis and prognosis of incurable cancer would have negative effects on the patients’ emotional well-being and would contribute to the deterioration of the patients’ condition. The caregivers wished to encourage the patients rather than discourage them with unfortunate news.

*‘No. I have not talked to him. He is still good and does not know what he has. I do not want him to know and do not want to discourage him. I encourage him. I can simply say that I do not want him to know what he has’.*

(P4, wife, caregiver of a 51-to-60-year-old man with colon cancer, Islam)

***Theme 2: Communication patterns between caregivers and patients***

Two common communication patterns were identified among the caregivers. The caregivers avoided discussing the advanced stage of cancer. In certain situations, they provided the patients with unreliable information.

*Sub-theme 2.1: Avoiding to say that word*

The caregivers did not inform the details of the disease and treatments when the patients did not ask. Otherwise, information about the symptoms and signs was communicated instead of the diagnosis and prognosis.

*‘He takes medicines as prescribed. I do not tell what the medicines are. He is good about taking them every time and never refuses, and it has never been a problem. He is easy-going. This is one of his good qualities. He has never made me feel bad’.*

(P1, daughter, caregiver of an 81-to-90-year-old man with lung cancer, Buddhism)

*‘He used to ask. I told him it was a painkiller. I did not say it was morphine’.*

(P5, daughter, caregiver of an 81-to-90-year-old man with lung cancer, Islam)

*Sub-theme 2.2: It is something else*

One strategy that the caregivers used in communicating with the patients was to distort the truth.

*‘Did not tell all. I told her that it was an abscess’.*

(P3, daughter, caregiver of a 91-to-100-year-old woman with kidney cancer, Islam)

The caregiver of a patient who experienced constipation informed the patient that his symptoms were due to haemorrhoids rather than advanced-stage rectal cancer.

*‘Constipation. I told him haemorrhoids’.*

(P7, daughter, caregiver of an 81-to-90-year-old man with rectal cancer, Islam)

***Theme 3: Difficulties and challenges in maintaining concealment***

The concealment of the diagnosis and prognosis of advanced-stage cancer presented communication challenges. While most caregivers believed it was the best option, some caregivers faced difficulties and challenges. Furthermore, concealing the information or maintaining the conspiracy of silence was sometimes unsuccessful. Some caregivers suspected that the patients might be aware of their advanced-stage cancer. However, communication between the caregivers and patients remained vague.

*Sub-theme 3.1: Feeling guilty about hiding the truth*

One caregiver expressed that concealing the information was a source of guilt for her. However, the decision to keep the information a secret was made by the whole family.

*‘Yes, I looked at her and knew what she was. Sometimes, she suffered from pain, and I cried in front of her. She asked me if her deteriorating condition was just part of the ageing process, why I was sad and why I did not allow her to die. Now, I can partially deal with this. There are two conflicting feelings on my mind’.*

(P8, granddaughter-in-law, caregiver of an 81-to-90-year-old woman with lung cancer, Buddhism)

*Sub-theme 3.2: Should I unveil the secret (or not)?*

Some caregivers were unsure about whether they should disclose the truth or not.

*‘If the day comes... If the symptoms are..., I will tell him’.*

(P10, son, caregiver of a 71-to-80-year-old man with leukaemia, Buddhism)

*Sub-theme 3.3: (S)he might have known already*

Concealing information about the diagnosis and prognosis or maintaining the conspiracy of silence may not always prevent the truth from emerging. Patients may have suspicions and might become aware of their conditions through other sources of information.

*‘Anyway, she thinks about it. She has no idea when she will go [die]’.*

(P3, daughter, caregiver of a 91-to-100-year-old woman with kidney cancer, Islam)

*‘He knows, I think. Some people came and visited him, and they talked. My father closed his eyes; he was listening’.*

(P5, daughter, caregiver of an 81-to-90-year-old man with lung cancer, Islam)

***Theme 4: Communication support from healthcare professionals***

Both concealing and disclosing the information required communication support from healthcare professionals in different ways.

*Sub-theme 4.1: Please keep a secret*

The caregivers needed healthcare professionals to avoid informing the patients about the diagnosis and prognosis.

*‘Well, there is no need [for healthcare professionals] to talk much. Taking medications and measuring blood pressure can be done as usual. Old people are worried, even if they do not have any disease’.*

(P6, daughter, caregiver of an 81-to-90-year-old woman with rectal cancer, Islam)

*Sub-theme 4.2: It is okay to tell (the diagnosis and prognosis) later*

Some caregivers were unsure if concealing the information was beneficial for the patients. Healthcare professionals could serve as a source of support for making decisions and providing information to patients. A caregiver would follow the advice of a physician on whether the diagnosis and prognosis should be disclosed or not.

*‘In my mind, I am afraid that she will regret. It depends on your [the interviewer – a physician] consideration. But I suddenly feel doubtful. It is hopeless when the treatment is lengthy’.*

(P3, daughter, caregiver of a 91-to-100-year-old woman with kidney cancer, Islam)

## Discussion

This study revealed four themes related to communication issues regarding the concealment of advanced-stage cancer between caregivers and patients. First, the patients’ personality, characterised by anxiety and overthinking, and concerns about negative effects on emotions and deteriorating conditions were the reasons for concealing information about the diagnosis and prognosis. Second, two communication patterns between the caregivers and patients were observed: communicating symptoms and signs instead of the diagnosis and prognosis and distorting information about the diagnosis and prognosis. Third, the caregivers’ feelings of guilt, hesitation in disclosing information about advanced-stage cancer and suspicion of the patients’ awareness of advanced-stage cancer constituted the difficulties and challenges in communication. Lastly, healthcare professionals could support communication by refraining from informing patients about the diagnosis and prognosis and by assisting in making decisions and disclosing the information.

In this study, all caregivers considered concealing information about the diagnosis and prognosis to prevent negative effects on the patients’ well-being. Nevertheless, a systematic review revealed the inconclusive effects of prognostic disclosure on patients’ quality of life.^11^ In terms of the quality of communication, information concealment or the conspiracy of silence is often considered a communication failure in palliative care.^14^ Concealing information about cancer is a common practice in some cultures and beliefs where the term ‘cancer’ is considered taboo.^15-18^ Based on this belief and the anticipation of negative effects, the caregivers in this study communicated with the patients by distorting the information to avoid delivering unfortunate news. Subsequently, some communication difficulties and challenges arose among the caregivers.

Concealing information not only affects communication between caregivers and patients but also causes unfavourable impacts on healthcare professionals, such as impaired relationships with patients, emotional burden, ethical issues and a lack of an appropriate transition from curative care to palliative care.^13^ Providing reliable and sufficient information about the disease to patients is expected in several settings, especially in Anglo-Saxon countries.^25^ In the Thai context, information concealment is a matter of debate due to the differing perspectives between cultural norms and palliative care practices that adopt core concepts from western countries. The findings of this study indicated that caregivers may reconsider their decision to disclose the information in the future. A study conducted in Thailand revealed that family members changed their perspectives to be more positive after the disclosure of information.^26^ However, some families maintained their decision to conceal the information.^26^

This study reflected the communication issues from the caregivers’ perspectives. It was assumed that the patients were not aware of the actual information. Based on this communication pattern, the patients were not at the centre of the care process. From healthcare providers’ perspectives, this was considered a communication failure.^14^ In contrast, most caregivers did not recognise this as a problem because they wished to protect the patients from unfavourable situations. Decisionmaking was the responsibility of the families rather than the patients.^27^ This discordance between healthcare providers and families may be prevented through honest communication from the beginning of the care process.^14^ Once information concealment or the conspiracy of silence occurs, exploring the root causes with respect for patients’ and family’s beliefs is recommended.^14^

Based on the authors’ 6-year experience in establishing palliative care services in a district hospital, the concealment of information about advanced-stage cancer used to be common. It was one of the most challenging issues in the authors’ palliative care practice. In this study, only 10 patients who received palliative care consultations were identified throughout the study period. This suggests that palliative care consultations may increase awareness of the disease diagnosis and prognosis among patients and caregivers. However, information concealment continues to persist in the authors’ practice. Therefore, healthcare professionals and palliative care teams should prepare themselves to address communication issues in positive and supportive ways when dealing with the concealment of advanced-stage cancer.

This study has two main strengths. First, although palliative care has been emphasised as an essential health service in Thailand, its understanding among the general public remains limited. This study was conducted in a district hospital where palliative care services cover outpatient, inpatient and home care. The findings reflected communication issues across both hospital and non-hospital settings. Second, the participants of this study were the caregivers and family members of the patients. Non-family caregivers or private caregivers in commercial settings were excluded. These inclusion and exclusion criteria helped in understanding the background of the families’ decisions. Despite these strengths, three study limitations must be considered. First, the qualitative study design limited the generalisability of the findings. Second, the small number of participants might have restricted the understanding of various communication patterns. Nevertheless, data saturation was achieved. Third, the participants were caregivers, which limited the understanding of patients’ perspectives on communication issues.

In conclusion, this study highlights the reasons for concealing advanced-stage cancer among caregivers, including communication patterns and challenges and the need for supportive communication from healthcare professionals. Communicating information about advanced-stage cancer can change over disease progression and care processes. Healthcare professionals can support communication throughout care if families wish to disclose the diagnosis and prognosis to patients. Although information concealment involves both caregivers and healthcare providers, patients remain at the centre of care. Future studies should focus on decisionmaking and communication processes to enhance communication strategies when addressing information concealment, ultimately benefitting patients.

## Data sharing statement

Data are available upon reasonable request.

**How does this paper make a difference in general practice?**

- The concealment of advanced-stage cancer is considered an appropriate communication approach by some families to prevent unpredictable negative effects on patients’ well-being.
- Concealing information about the diagnosis and prognosis leads to unreliable communication and communication challenges.
- The concealment of advanced-stage cancer can evolve over time, and healthcare professionals can support communication during both concealment and disclosure of the information.
